# Investigating the Anticancer Potential of Salvicine as a Modulator of Topoisomerase II and ROS Signaling Cascade

**DOI:** 10.3389/fonc.2022.899009

**Published:** 2022-06-01

**Authors:** Dipta Dey, Mohammad Mehedi Hasan, Partha Biswas, Stavros P. Papadakos, Rehab A. Rayan, Sabiha Tasnim, Muhammad Bilal, Mohammod Johirul Islam, Farzana Alam Arshe, Efat Muhammad Arshad, Maisha Farzana, Tanjim Ishraq Rahaman, Sumit Kumar Baral, Priyanka Paul, Shabana Bibi, Md. Ataur Rahman, Bonglee Kim

**Affiliations:** ^1^Biochemistry and Molecular Biology department, Life Science Faculty, Bangabandhu Sheikh Mujibur Rahman Science and Technology University, Gopalgonj, Bangladesh; ^2^Department of Biochemistry and Molecular Biology, Faculty of Life Science, Mawlana Bhashani Science and Technology University, Tangail, Bangladesh; ^3^Department of Genetic Engineering and Biotechnology, Faculty of Biological Science and Technology, Jashore University of Science and Technology (JUST), Jashore, Bangladesh; ^4^ABEx Bio-Research Center, East Azampur, Dhaka, Bangladesh; ^5^First Department of Pathology, School of Medicine, National and Kapodistrian University of Athens (NKUA), Athens, Greece; ^6^Department of Epidemiology, High Institute of Public Health, Alexandria University, Alexandria, Egypt; ^7^Department of Pharmaceutical Chemistry, Faculty of Pharmacy, University of Dhaka, Dhaka, Bangladesh; ^8^College of Pharmacy, Liaquat University of Medical and Health Sciences, Jamshoro, Pakistan; ^9^Department of Biochemistry and Microbiology, North South University, Dhaka, Bangladesh; ^10^College of Medical, Veterinary and Life Sciences, University of Glasgow, University Avenue, Glasgow, United Kingdom; ^11^Department of Biotechnology and Genetic Engineering, Faculty of Life Science, Bangabandhu Sheikh Mujibur Rahman Science and Technology University, Gopalganj, Bangladesh; ^12^Microbiology department, Jagannath University, Dhaka, Bangladesh; ^13^Yunnan Herbal Laboratory, College of Ecology and Environmental Sciences, Yunnan University, Kunming, China; ^14^Department of Biological Sciences, International Islamic University, Islamabad, Pakistan; ^15^Global Biotechnology & Biomedical Research Network (GBBRN), Department of Biotechnology and Genetic Engineering, Faculty of Biological Sciences, Islamic University, Kushtia, Bangladesh; ^16^Department of Pathology, College of Korean Medicine, Kyung Hee University, Seoul, South Korea; ^17^Korean Medicine-Based Drug Repositioning Cancer Research Center, College of Korean Medicine, Kyung Hee University, Seoul, South Korea

**Keywords:** diterpenoid quinone, multidrug-resistant (MDR), topoisomerase II, ROS signaling, DNA damage response (DDR), anticancer properties

## Abstract

Salvicine is a new diterpenoid quinone substance from a natural source, specifically in a Chinese herb. It has powerful growth-controlling abilities against a broad range of human cancer cells in both *in vitro* and *in vivo* environments. A significant inhibitory effect of salvicine on multidrug-resistant (MDR) cells has also been discovered. Several research studies have examined the activities of salvicine on topoisomerase II (Topo II) by inducing reactive oxygen species (ROS) signaling. As opposed to the well-known Topo II toxin etoposide, salvicine mostly decreases the catalytic activity with a negligible DNA breakage effect, as revealed by several enzymatic experiments. Interestingly, salvicine dramatically reduces lung metastatic formation in the MDA-MB-435 orthotopic lung cancer cell line. Recent investigations have established that salvicine is a new non-intercalative Topo II toxin by interacting with the ATPase domains, increasing DNA–Topo II interaction, and suppressing DNA relegation and ATP hydrolysis. In addition, investigations have revealed that salvicine-induced ROS play a critical role in the anticancer-mediated signaling pathway, involving Topo II suppression, DNA damage, overcoming multidrug resistance, and tumor cell adhesion suppression, among other things. In the current study, we demonstrate the role of salvicine in regulating the ROS signaling pathway and the DNA damage response (DDR) in suppressing the progression of cancer cells. We depict the mechanism of action of salvicine in suppressing the DNA–Topo II complex through ROS induction along with a brief discussion of the anticancer perspective of salvicine.

## Introduction

Cancer is a menace to humankind that causes millions of deaths worldwide every year (1). Cancer occurs when there is an uncontrollable cell division in any tissues of a living body, and it usually proliferates, causing the invasion of other healthy tissues in an individual. It is still one of the most challenging issues in the medical and public health sectors (2–4). The American Cancer Society evaluates cancer cases annually in the USA, and its most recent estimate in 2021 revealed 1,898,160 cases with 6,08,570 mortalities (5–8). Furthermore, a lot of conditions are associated with the most concerning risk factors for cancers, notably age, sex, genetic and/or epigenetic factors, coexisting complications, and so forth (9, 10). Smoking, pollution, and obesity affect the rate of lung cancer and cancers related to reproductive organs, such as prostate, ovarian, cervical, and breast cancers (11–14). Likewise, other risk factors such as solar exposure, lousy diet, and radiation exposure affect cancers of the stomach, colon, liver, pancreas, kidney, and bladder, as well as glioblastoma and skin cancers. Additionally, polluted air with benzene can trigger some effects on leukemia, lymphoma, and myeloma (15). As maintained by the National Cancer Institute, breast cancer in women is the most common cancer type (approximately 2.3 million new cases occur annually), prostate the second, and the third is lung cancer. Colorectal cancer takes the fourth position in the survey; however, liver and intrahepatic bile duct cancers have the least number of new cases (16, 17). As a matter of fact, mutations in the *BRCA1*/*BRCA*2 genes stimulate early-onset breast cancer even if patients do not have any family history of cancer (18, 19). The World Cancer Research Fund claimed that the consumption of healthy foods could significantly reduce cancer risks, and some diets like the Mediterranean diet can reduce the severity of gastric, glioblastoma, colon, rectal, bladder, liver, and lung cancers. Notably, a high intake of roughage is known to minimize the risk of colorectal cancers (20). Multiple phytocompounds, without any doubt, have been applied in an anticancer perspective and have shown positive outcomes in clinical trials, along with acting as potential anticancer agents (21–24).

Salvicine is a potential bioactive phytomolecule belonging to the diterpenoquinone derivatives extracted from *Salvia prionitis* Hance, a Chinese herbal medicinal plant. It has a chemically active quinone moiety and imparts antitumor activity by modulating the generation of reactive oxygen species (ROS) (25). However, salvicine directly acts toward topoisomerase II (Topo II), or simply Topo II enzyme, as Topo II is especially crucial in regulating the DNA topology, notably DNA replication, transcription, recombination, and cell cycle (26, 27). Type II topoisomerases include two main subdivisions—notably Topo IIα and Topo IIβ—and hence human cells express such enzymes from Topo IIα and Topo IIβ genes, respectively (28). However, Topo IIα plays a key role in the cell division stage of the S and M phases of the cancer cell cycle at the same time as its role in both DNA multiplication and chromosome separation for mitotic cell division. Conversely, Topo IIα is not related to cell sustainability, sometimes trapped by transcriptional factors of multiple cancerous cells; therefore, it is not related to cell division (29, 30). Importantly, Topo II is the principal drug target site for cancer treatment *via* following its anti-proliferative activity, DNA double-strand breaks (DSBs), and inducing apoptosis-related pathways (31, 32). Accordingly, salvicine effectively elevates the level of intracellular ROS, which potentially initiates the DSBs through the *N*-acetylation of cysteine (NAC) residues (33). Multiple scientific reports have demonstrated that salvicine upregulates the level of ROS in the cellular system, which subsequently blocks the enzyme activity of Topo II and mediates DNA DSBs, apoptosis, and the cytotoxicity mechanism (34–36).

Additionally, salvicine mediates the activation of the following pathways: ataxia telangiectasia mutated (ATM) kinase, ataxia telangiectasia and Rad3-related (ATR) kinase, and histone H2AX; therefore, the complete experimental trail occurs in lung carcinoma A549 cells (37). It is noteworthy that it directly shuts off the activity of the telomerase enzyme and subsequently inhibits lung carcinoma, as well as the initiation of apoptotic pathways, as shown in a research work on the A549 and HL-60 cell lines. (33, 38). Salvicine causes genomic DNA DSBs in HL-60 human promyelocytic leukemia cells and MCF-7 breast cancer cells (39). It shows activity toward not only several cancer cases but also in different cell lines including K562, HL-60, U-937, K-562, HL-60, A02, SGC-7901, MKN-28, A549, SPC-A4, NCI-H23, NCI-H522, S-180, MCF-7, and HeLa. All these are shown in detail in **Table 1**. In leukemia, this novel compound follows diverse pathways, among them are enhancement of cytotoxic activity, induction of the apoptotic pathway, initiation of promoters mediating DNA damage, reduction of the telomerase enzyme activity, decrease of *mdr-1 via* blocking Topo II, repression of the activation of the transcription factor *c-Jun*, and production of the ROS moiety involved in cell death with P-glycoprotein (P-gp) activity reduction (58–64). At the same time, its cytotoxic activity has been proven in several cell lines, such as in K562, HL-60, U-937, K-562, A02, SGC-7901, and MKN-28, with such activity being more dose-dependent. In contrast, salvicine is involved in anti-stomach cancer activity by following cytotoxicity, apoptotic, and antitumor mechanisms. It is important to note that the experiment was not only performed on human patients but also involved the different cell lines mentioned, i.e., SGC-7901 and MKN-28 (64, 65). Several scientific reports have demonstrated that the A549, SPC-A4, NCI-H23, and NCI-H522 cell lines also showed more positive results in lung carcinoma after the administration of salvicine at specific doses *via* following mechanisms such as telomerase inhibition, decreased *p53* gene levels, telomeric protein TRF2 damage, and anti-angiogenic activity (66–69). Additionally, the MCF-7, ADR, and MDA-MB-435 cell lines were used for the analysis of the anti-breast cancer activity of salvicine. Moreover, this compound maintains diverse mechanisms including DNA double-strand damage, apoptosis, derangement of cell adhesion to the extracellular matrix (ECM), and cytotoxic activity (61, 70–72). Other more authentic scientific reports have stated that salvicine has more anticancer activity in sarcoma, pancreatic cancer, cervical cancer, and oral carcinoma (61, 65, 73, 74). In tumor metastasis, salvicine influences the cell adhesion genes and decreases the expressions of different integrin proteins, i.e., integrin a3/a6/aE/b3/b5/b8, paxillin, and focal adhesion kinase (FAK), in human breast cancer; its anti-metastatic efficiency has also been suggested (75). On the other hand, it elicits the activation of extracellular signal-regulated kinase (ERK) and p38 mitogen-activated protein kinase (p38 MAPK). It has also shown effects on integrin-mediated cell adhesion in U0126 and SB203580, as well as on the inhibition of both p38 MAPK and MAPK/ERK (55). Salvicine remarkably reduces the expression level of the Rho protein, mainly RhoC in primary tumors (without affecting RhoA), and it significantly disrupts the Rho-dependent stress fiber and subsequently restricts the cell adhesion and motility, also downregulating the gene expression along with control of functional proteins such as fibronectin, integrins, FAK, RhoC, and paxillin, which are associated with these pathways (75). Another research group showed that salvicine decreases the level of RhoA (GTP-mediated) and damages actin stress fiber networks crucial to cell adhesion *via* ROS-related inhibition of the activity of RhoA (55). Salvicine also possesses cytotoxic properties against a variety of multidrug-resistant (MDR) tumor cells *via* downregulating P-gp expression. It suppresses the expression of the *mdr-1* gene, and hence P-gp expression, by stimulating the gene expression of mainly c-Jun in MDR K562/A02 cells.

**Table 1:** Potential activities of salvicine in numerous cancer types and cancer cell lines with their significant mechanisms of actions.

Cancer type	Cell line	Mechanism of action	Reference
Leukemia	K562	Cytotoxic effects	(40)
		Dose- and time-dependent fixation in the G1 phase	
	HL-60	Cytotoxic effects	
	K562	Dose- and time-dependent fixation in the G1 phase	(41)
		Induction of apoptosis	
	HL-60	Cytotoxic effects	(42)
		DNA double-strand breaks in the *c-myc* P2 promoter	
		Induction of apoptosis	
		Decreased *c-myc*, increased *c-Fos* and *c-Jun*	
	HL-60	Dose- and time-dependent decrease in telomerase activity	(38)
		Upregulation of protein phosphatase 2A (PP2A)	
	K562	Upregulation of cytotoxicity	(43)
		Decreased apoptosis	
	K562/A02	Cytotoxic effects	(44)
		Stimulation of caspase-1 and caspase-3	
		Enhancement of the Bax/Bcl-2 ratio	
		Downregulation of Bcl-2	
		Decreased P-gp expression	
	K562	Decreased *mdr-1* gene in MDR cell lines	(36)
		Increased c-Jun expression in MDR and K562 cell lines	
		Increased phosphorylation of c-Jun and JNK in MDR and K562 cell lines	
	K562	Production of ROS in K562 and MDR cell lines	(45)
		GSH exhaustion in K562 and MDR cell lines	
		H_2_O_2_ scavengers and NAC inhibit the cell toxicity of salvicine	
		H_2_O_2_ and vitamin C induce salvicine-mediated cell toxicity and apoptosis in K562 and MDR cell lines	
		Catalase reverses the effects of H_2_O_2_ and vitamin C	
		NAC inhibits salvicine-mediated: P-gp downregulation, JNK phosphorylation, and c-Jun potentiation	
Stomach cancer	SGC-7901	Cytotoxic effects	(46)
	MKN-28	Cytotoxic effects	
	SGC-7901	Dose- and time-dependent fixation in the G1 phase	(47)
		Increased apoptosis	
	SGC-7901	Decreased cell growth	(48)
Lung cancer	A549	High dose, short duration: decreased telomerase activity	(49)
		Low dose, long duration: telomere shrinkage, decreased telomerase activity	
	A549	Decreased p53 levels	(50)
		Modified expression of p53-related genes (*Bax* and *mdm2*)	
		Not a substrate of P-gp	
		Decreased growth rate	
		Enhanced mobility	
		Types of pathology: double-strand breaks, telomere DNA damage, telomere contraction	
		TRF2 disintegration	
	A549 cells	ATR: responsible for telomeric damage	(51)
		TRF2 downregulation activates ATR; ATR downregulation prevents TRF2 downregulation after salvicine incubation	
	A549 cells	Cytotoxic effects	(46)
	SPC-A4	Cytotoxic effects	
	NCI-H23	Cytotoxic effects	
	NCI-H522	Cytotoxic effects	
	A549	Decreased cell growth	(52)
	HMEC	Decreased movability of HMECs	
		Downregulation of the microtubule formation of HMEC	
		Double IC_50_ on A549	
Sarcoma	*In vivo* S-180	Tumor shrinkage	(48)
Breast cancer	MCF-7	DNA double-strand damage	(53)
		Salvicine-induced *c-myc* damage	
		Downregulated p53, p53-independent apoptosis	
		Increased *c-Jun* expression	
	MCF-7	ROS formation	(54)
		DNA double-strand breaks	
		Damage amelioration by antioxidants	
		Reversal of DNA damage by heat	
		Negative regulation of DNA-PK reversed by antioxidants	
		NAC-induced apoptosis and cell toxicity reversal	
	MDA-MB-435	Derangement of cell adhesion to the extracellular matrix	(55)
		Cytoskeleton abnormalities: round cell morphology	
		Decreased integrin beta-1 ligand affinity	
		Induction of the MAPK/ERK pathway	
		Induction of ROS formation	
	MCF-7/ADR and MCF-7	Cytotoxic effects	(44)
Pancreatic cancer	SW1990-GEM	Downregulation of the *mdr-1* gene (in toxic concentrations)	(56)
Cervical cancer	HeLa	H_2_O_2_ generation precedes DNA double-strand breaks and apoptosis	(57)
		Salvicine inhibits GSH	
		Catalase and the insertion of GSH antagonize the effects of salvicine on DNA and apoptosis	
	altered Topo II HL-60/MX2 cells	Resistance to salvicine-mediated DNA damage	
Oral carcinoma	KB/VCR and KB	Cytotoxic effects	(44)

MDR, multidrug-resistant; ROS, reactive oxygen species; ATR, ataxia telangiectasia and Rad3-related; JNK, c-Jun N-terminal kinase; NAC, N-acetyl cysteine; HMECs, human microvascular endothelial cells; DNA-PK, DNA-dependent protein kinase; GSH, glutathione; MAPK, mitogen-activated protein kinase; ERK, extracellular signal-regulated kinase.

The studies included in the current review specifically emphasize that salvicine potentially blocks the DNA Topo II enzyme, which is an interesting target for cancer treatment, along with diterpenoquinone compounds that impart the ROS molecule and are directly involved in DNA double-strand damage, DNA DSBs, apoptosis, and cytotoxicity mechanism. Furthermore, we included here multiple anticancer perspectives of salvicine from published scientific reports.

## Overview of Salvicine

Salvicine is a new diterpenoid quinone compound that is produced by modifying the composition of a natural product lead found in the Chinese herb *S. prionitis* Hance (Labiatae) (76). It was chemically synthesized in 1999 by Sheng et al. and has been demonstrated to have substantial inhibitory effects against a broad spectrum of human tumor cells both *in vitro* and in mice harboring human tumor xenografts (77, 78). Specifically, salvicine and its analogs are non-intercalative Topo II inhibitors that have demonstrated potent anticancer efficacy both *in vitro* and *in vivo*, as well as a broad spectrum of anti-MDR activities in animal models (79). It has been reported that salvicine causes the breakage of two strands of DNA by promoting Topo II activity and blocking re-ligation, which is correlated with the suppression of tumor growth. Salvicine also promotes apoptosis in human cancer cells *via* inducing DNA damage to specific genes. It has also been shown that salvicine possesses anticancer activity manifested in a variety of ways, including downregulation of Topo II, DNA damage, bypassing multidrug resistance, and prevention of tumor cell adhesion (78). It has been revealed that a new family of salvicine compounds exhibited significant cytotoxicity against tumor cell lines (80). Salvicine and its derivatives showed significant activity against solid tumor cells, particularly lung and gastric cancer cells, in comparison to its modest cytotoxicity on leukemia cell lines (46). *In vivo* experiments revealed that salvicine exhibited strong anticancer efficacy against murine S-180 sarcoma and Lewis’s lung cancer, as well as in human lung adenocarcinoma xenograft models, in addition to other cancer types (48). Furthermore, a study found that the anticancer activity salvicine was correlated with its ability to cause tumor cell apoptosis with comparable potency against both human leukemia cells and gastric carcinoma cells, showing its particular impact on solid tumor cells (47). Three cell lines (K-562/A02, MCF-7/ADM, and MKN28/VCR) were found to be resistant to a lot of different drugs, but the cytotoxicity of salvicine was not affected by P-gp. These research findings indicate that salvicine is a promising anticancer drug. It is currently undergoing clinical trials in several countries (33).

## ROS Signaling Pathway, DNA Damage Response, and Cancer

ROS are a collection of transient elements initially delineated as free radicals in skeletal muscle, such as O_2_, OH, and H_2_O_2_ (81). They were first thought of as dangerous derivatives of respiration by the mitochondria, but recent breakthroughs have shed light on the cellular operational functions of ROS from meliorating immunity, e.g., oxidative changes in scavenger cells, to getting rid of microorganisms and to acting as signaling elements, such as H_2_O_2_ modulating the MAPK and NF-κB pathways (82, 83). ROS are produced internally *via* the mitochondria, with O_2_ acting as the ultimate electron recipient for electron transmission series (84); NADPH oxidase, a cellular membrane-linked enzyme (85); peroxisomes, which contain enzymes for the production of H_2_O_2_, such as polyamine oxidase (86); and the endoplasmic reticulum, which induces H_2_O_2_ as a derivative in folding peptides or during vulnerability to exogenic tension such as chemotherapy, ionizing radiation (IR), or ecological breaches that impact the previously mentioned cell organs and enzymes (87). The production of ROS has connections with the outcomes of radiation therapy or chemotherapy through its impacts on backward cellular viability or fatality rate ranges (88, 89). Therefore, ROS modulators could be employed in malignant neoplasm primary prevention or to intensify treatment outcomes (90). There have been trivial advancements in the understanding of ROS from the research laboratory to the medical institution (40). For instance, despite the auspicious *in vitro* data, some antioxidant trials in the prevention of malignant neoplasm have shown contradictory findings, highlighting the demand for further understanding of such procedures in cells (91).

**Figure 1:**
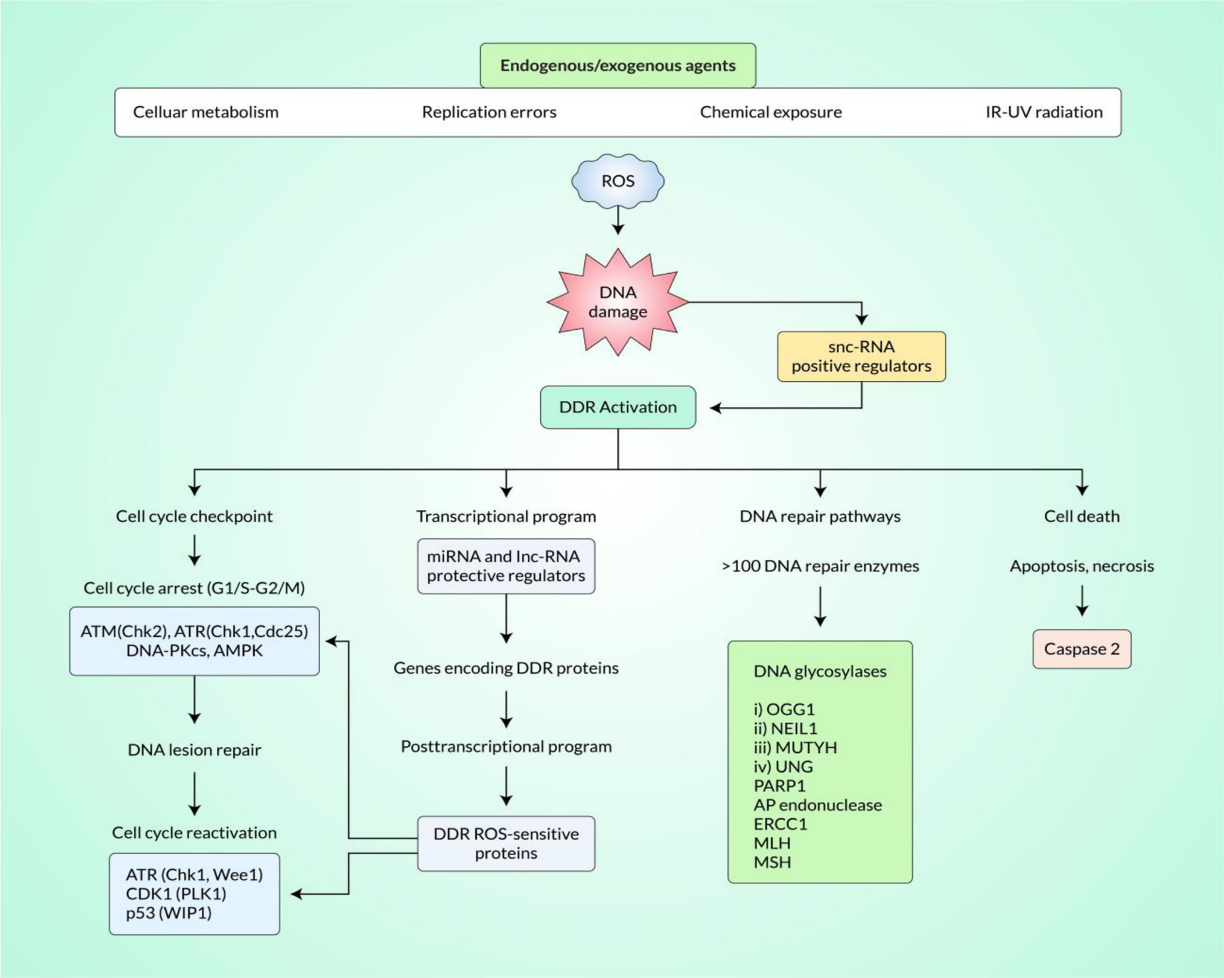
Induction of DNA damage response (DDR) by reactive oxygen species (ROS) signaling in cancer tissue. ROS signaling pathways can effectively induce the DDR in cancer tissue. Small non-coding RNA (snc-RNA) positive regulators play important roles in DDR activation. DDR activation effectively modulates the cell cycle checkpoint, transcriptional program, DNA repair pathways, and programmed cell death.

DNA impairment denotes chemical or physical alterations of cellular DNA, which could impact the reading and sending of inherited data. DNA can be altered by a variety of endogenic and exogenic injuries involving radiation, chemicals, free radicals, and topologic alterations, all of which stimulate well-defined types of impairment (92). Cells have developed composite procedures for managing impairments to the genome. Regarding the reason for injury to the DNA, certain pathway/s are triggered for ease of determining the altered parts and their repair (93). Indeed, DNA DSB is a serious mutagenic injury because of the arrangements inside the chromosomes or failure of hereditary data for the wrong DNA repair. As a reaction to DNA impairment, a system of events jointly called DNA damage response (DDR) is triggered (**Figure 1**). Such an effect involves identifying the DNA impairment, stimulating checkpoints, halting the cellular processes, and, finally, the processes of repair, apoptosis, and immune clearance (94–96). The building blocks of DSB-generated DDR have been analyzed and sorted into three main units: sensors to determine the impairment, transducers to organize signaling, and effectors to coordinate final events (97). Different DNA impairment outcome/repair pathways involve mismatch repair for incompatible bases, nucleotide excision repair (NER) for the cross-linking of intra-strand thymidine dimers, base excision repair (BER) for base alterations, single-strand annealing for single-strand DNA (ssDNA) damage, and transcription-coupled repair (TCR) for transcription-mediated impairments. However, the DDR motivated by DSBs triggers a system of similar pathways involving homologous recombination (HR), non-homologous end-joining (NHEJ), microhomology-mediated end-joining (MMEJ), and Fanconi anemia (FA) repair (93). The reaction to DSBs especially applies to carcinogenesis and chemotherapy, as many of the factors of the pathway are changed in malignant neoplasm, and so much of today’s radiotherapy and chemotherapy overwork such flaws (98).

### ROS in DNA Damage by Genotoxin

ROS have been recognized to mediate DNA impairment. For instance, IR stimulates DSBs *via* immediate great power impairment of the sugar pillar of DNA, besides *via* cellular free radicals, mainly the water’s OH. Chemotherapeutics such as cisplatin and doxorubicin increase the level of ROS, adding to their genotoxicity (99). ROS have likewise been denoted to instantly stimulate different types of DNA impairments *via* oxidizing nucleoside bases, for example, by producing 8-oxoguanine that could result in G–A or G–T transversions when unrestored (100). The BER pathway identifies and fixes oxidized bases; however, if they appear at the same time on opposing strands, BER could directly initiate DSBs (101). ROS generation likewise involves injuries, strand cracks, and decay of mitochondrial DNA (102).

### ROS-Mediated Oncogenic Stress in DNA Damage

The replication stress triggered by oncogenes is an influential origin of endogenous DNA damage and the production of DSBs in malignant neoplasms (103). Proto-oncogenes assist in cell proliferation and growth; however, overexpression or mutations could transform them into oncogenes, which causes continuous cellular development and carcinogenicity. Oncogenic cellular processes are linked to replication stress, which has been delineated as an abnormal replication fork series and formation of DNA (104). Replication stress finally leads to genetic imbalance and drives neoplasm evolution *via* the aggregation of additional pro-carcinogenic alterations (103). The DDR acts as a roadblock that confines the elaboration of unusual cellular replication; hence, it controls an exclusive force for carcinogenic-related DDR defects (105). Replication stress has diverse origins including abnormal source blasting, the action of physical obstacles, and disconnected DNA polymerase helicase from the replicating fork (104). The stimulation of oncogenes gives rise to ROS that affect replication stress events (106). dNTPs are oxidized by ROS to impact polymerase activity and, hence, decrease the speed of the replication forks *in vitro* (107). ROS can likewise impact replication fork advancement *via* the separation of peroxiredoxin 2 oligomers (PRDX2), where it creates a replisome-related ROS sensor that binds to the catalyst of the fork (TIMELESS). Increased ROS results in the separation of TIMELESS and PRDX2, therefore decreasing the replication fork velocity (108). Oxidized bases resulting from the activity of ROS likewise show physical barriers to replication forks (42), leading to the collapse of the replication forks at breakable positions along the genome, further resulting in DSBs and, finally, to over- or under-replicated DNA, with accompanying genomic imbalance in the neoplasm. The modulation of replication stress through ROS has medical indications, with various improved factors, such as the WEE1 and ATR inhibitors illustrated in **Figure 1**, focusing on replication stress in neoplasms (103).

### ROS in Detecting Double-Strand Breaks

**Figure 2:**
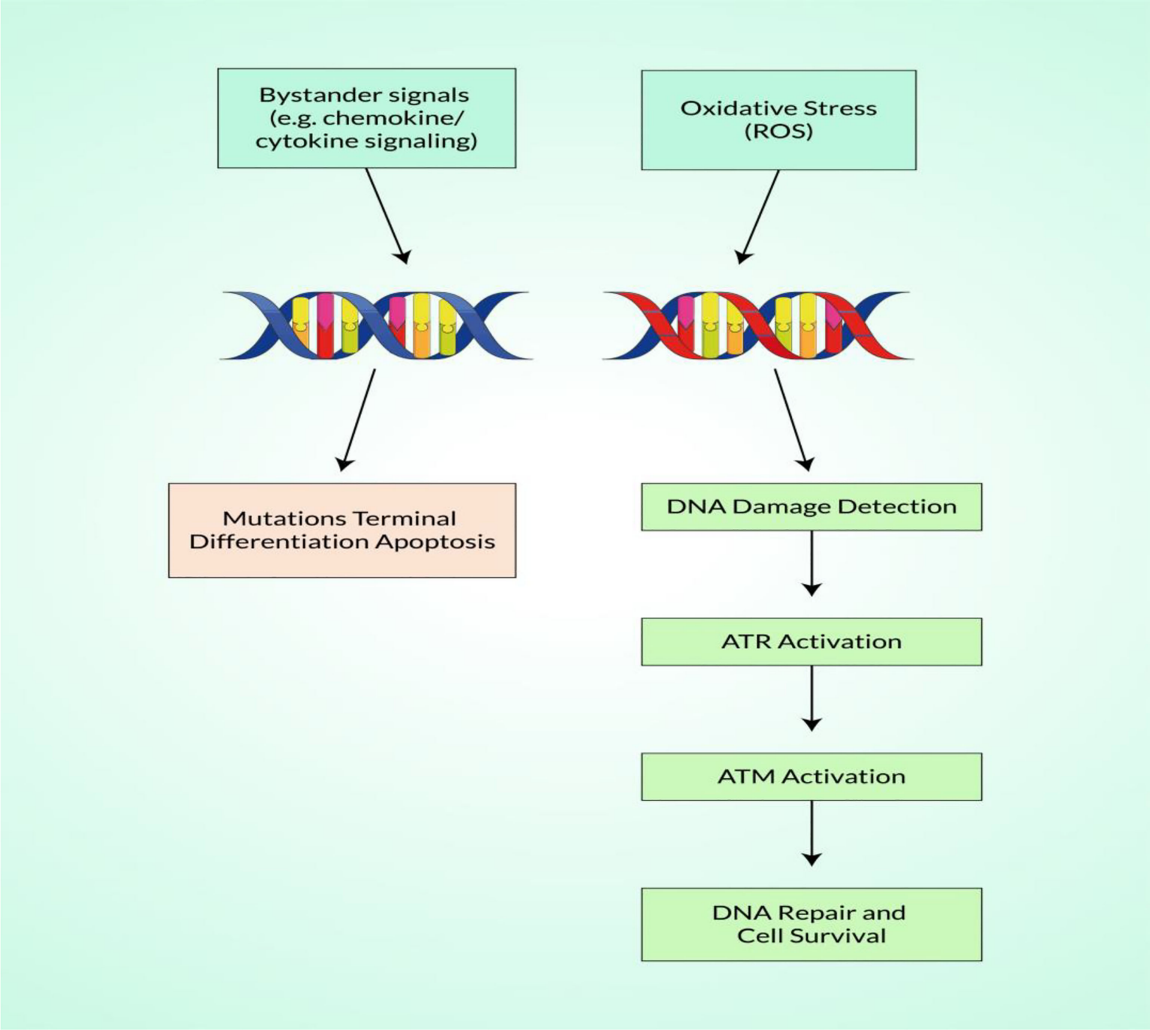
Schematic illustration of the detection of reactive oxygen species (ROS)-mediated DNA damage with DNA repair.

Generally, the DNA is continually harmed by both internal and external agents, such as chemicals and radiation. A DNA DSB is arguably the most important stage of damage because it can cause cell death if left unrepaired and chromosomal translocations if misrepaired, all of which are early steps in the etiology of carcinogenesis (109). DNA DSBs are unlikely to be directly caused to any significant degree by endogenously produced ROS, which mainly initiate base damage and single-strand breaks that are close to or during the reconstruction of other lesions (110, 111). In particular, DSB is the final lesion of a wide variety of DNA-damaging agents where ROS are not restricted to radical superoxide (O_2·_), hydrogen peroxide (H_2_O_2_), or hydroxyl radical (OH^−^) as endogenous DNA injury, including DSB (H_2_O_2_) (112). Importantly, base alteration is a typical ROS DNA injury, illustrated in **Figure 2**. Karanjawala et al. showed that ROS cause the most lethal form of DNA damage, DSBs (113). ROS can contribute to carcinogenic treatment *via* promotion of DNA damage, inactivation of the functions of key proteins, and insufficient regulation of specific cell growth regulators (114). A research study has revealed that, in comparison to healthy cells, the level of protein kinase CK-2 is higher in tumor cells. Researchers have also discovered that DMAT (2-dimethylamino-4,5,6,7-tetrabromo-1*H*-benzimidazole), unlike TBB (4,5,6,7-tetrabromo-1*H*-benzotriazole), induced ROS and DNA DSBs. Consequently, inhibition of the CK-2 enzyme, ROS, and DNA DSBs ultimately initiates the apoptotic pathway by DMAT (115). Many research studies have demonstrated that the activation of the STAT/MAP kinase/RAS-MAP kinase/PI3K pathways regulates the generation of ROS complexes. Some myeloid leukemias involve aggressive mutations in members of the RAS family in receptor tyrosine kinases (e.g., FMS-like tyrosine kinase receptor 3, FLT3) (NF-1) (116–118). These mutations collectively account for up to 50% of all acute myeloid leukemia (AML) cases and present leukemic cell proliferation and survival benefits by activating the genes that signal the RAS-MAP kinase, PI3K, and STAT pathways. FLT3 and internal tandem duplications (ITDs) enhance the production of ROS complexes (119, 120). In addition, approximately 30% of chronic carcinomas activate NRAS and KRAS mutations, and several laboratory studies have shown that they can modulate ROS and genomic instability (121, 122). Moreover, BCR–ABL tyrosine kinases are generated by the fusion of portions of BCR and ABL genes that initiate ROS development and a genomic instability cycle that can trigger DNA damage, especially DSBs in human carcinoma (41).

### ROS-Mediated Signal Transduction in DNA Damage Response and Potential Implications

Overexpression of ROS is closely related to the enhancement of the DDR response, which may be significant and positively related to genetic instability. DNA damage induces DNA oxidation, leading to a wide range of DNA changes, likely “indels,” i.e., insertions/deletions, base pair mutations, and DSBs, among which deletions and translocations are the most damaging DNA lesions (41, 123). Inadequate remediation of the DNA damage and DSBs by NHEJ and HR also contributes to genomic instability (114). Damages to the DNA are strictly regulated, and irregular function can significantly affect cells, leading to improvements in how cells can cope with genotoxic stress (95, 124, 125). The MRE11/RAD50/NBS1 (MRN), ATR-interacting protein (ATRIP), or Ku complexes, which activate ATM, ATR, and the DNA-dependent protein kinase catalytic subunit (DNA-PKcs), respectively, detect DNA DSBs, and several downstream proteins, including p53, are then targeted (126, 127). These damage sensors either provide the signals of the DNA repair pathways to repair the damage depending on the severity of DNA damage or induce cells to undergo apoptosis if the damage is excessive. At least two DSB repair processes, HR and NHEJ, are available. HR is an error-free repair route that involves RAD52, the end-binding DNA protein, and RAD51, which forms filaments across the unwound DNA strand to support the invasion. The resected 30 end is present in a homologous duplex of DNA and is expanded by DNA polymerase (124). NHEJ involves split DNA ends and minimal homology; this mechanism is not always accurate and can lead to small areas of non-model nucleotides around the DNA break site (microhomologies). NHEJ has two phases: a fast process that mixes basic DSB with compatible ends and a slower step that repairs DSB with complex non-compatible ends that require intervention (128).

Furthermore, new evidence has emerged for alternative end-to-end pathways that play significant roles in DSB repair but are less well defined. These routes lead to translocations and deletions coordinated by repair using significant microhomological DNA sequence levels and appear to be involved while the main NHEJ route remains missing or downregulated (129). In the absence of core NHEJ proteins, such as DNA ligase IV and Ku, Wang et al. reported that important end-joining in response to irradiation, and in the absence of Ku or DNA ligase IV, invalidates the end-joining results in translocations (130). In addition, although NHEJ is assumed to join the breakpoints of chromosomal translocations, alternative NHEJ will be the only primary mechanism of DNA end-joining in NHEJ-mutant mice and is very rare in humans with an NHEJ mutation (130). A research study revealed that the oxidative DNA damage in leukemia cells could lead to a wide range of DNA changes, including base pair mutations, insertions, and deletions (123).

**Figure 3:**
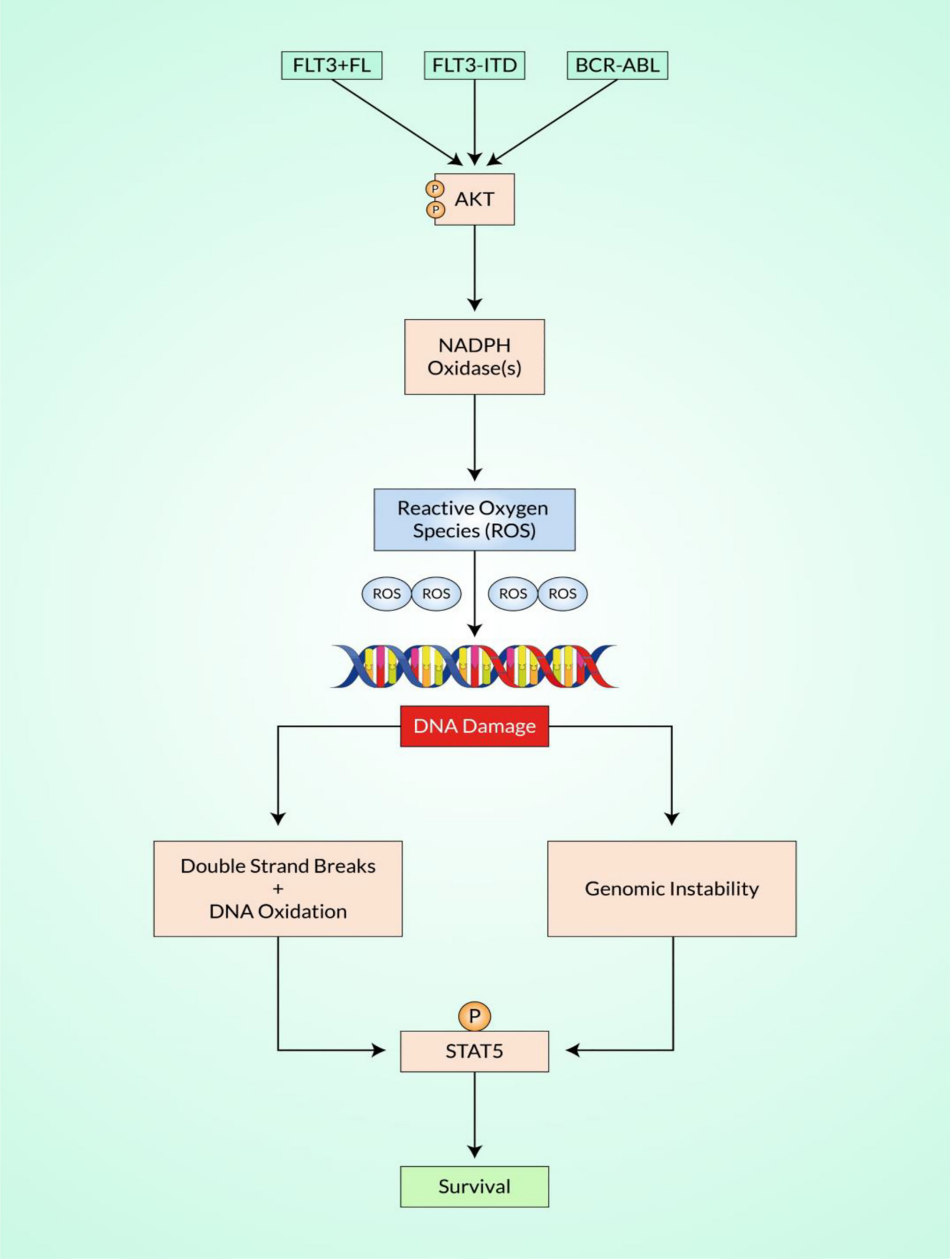
Schematic representation of reactive oxygen species (ROS)-mediated DNA damage response (DDR). Shown is a significant strategy in which the altered development of ROS increases the sources of endogenous DNA damage in various cancers, such as myeloid malignancies. In chronic myeloid leukemia (CML), the fusion gene *BCR*–*ABL* produces ROS, as do *FLT3*/*ITD* mutations in acute myeloid leukemia (AML) and RAS mutations in myelodysplastic syndromes (MDS)/myeloproliferative diseases (MPDs). Increased ROS can cause a sequence of genomic instabilities by Akt and NADPH oxidases, resulting in DNA double-strand breaks (DSBs) and altered repair, further leading to the acquisition of genomic modifications. There is accumulating evidence that defects in the primary signaling pathways for DSBs, non-homologous end-joining (NHEJ), and activation of the RAS/PI3K/STAT signaling pathways result in the increased expression of complementary or “backup” recovery, which can result in chromosomal deletions and translocations.

Increases in NHEJ activity and downregulation in the faithful NHEJ pathway have been observed in combination with oncogenic FLT3/ITD and BCR–ABL signaling (**Figure 3**) (131, 132). Therefore, further research has recently been done to explore particular mechanisms leading to the development of ROS and ROS-mediated damage in FLT3/ITD-positive AML cells to DNA and genome stability (133).

## Mechanism of Action of Salvicine in Inhibiting Topoisomerase II

**Figure 4:**
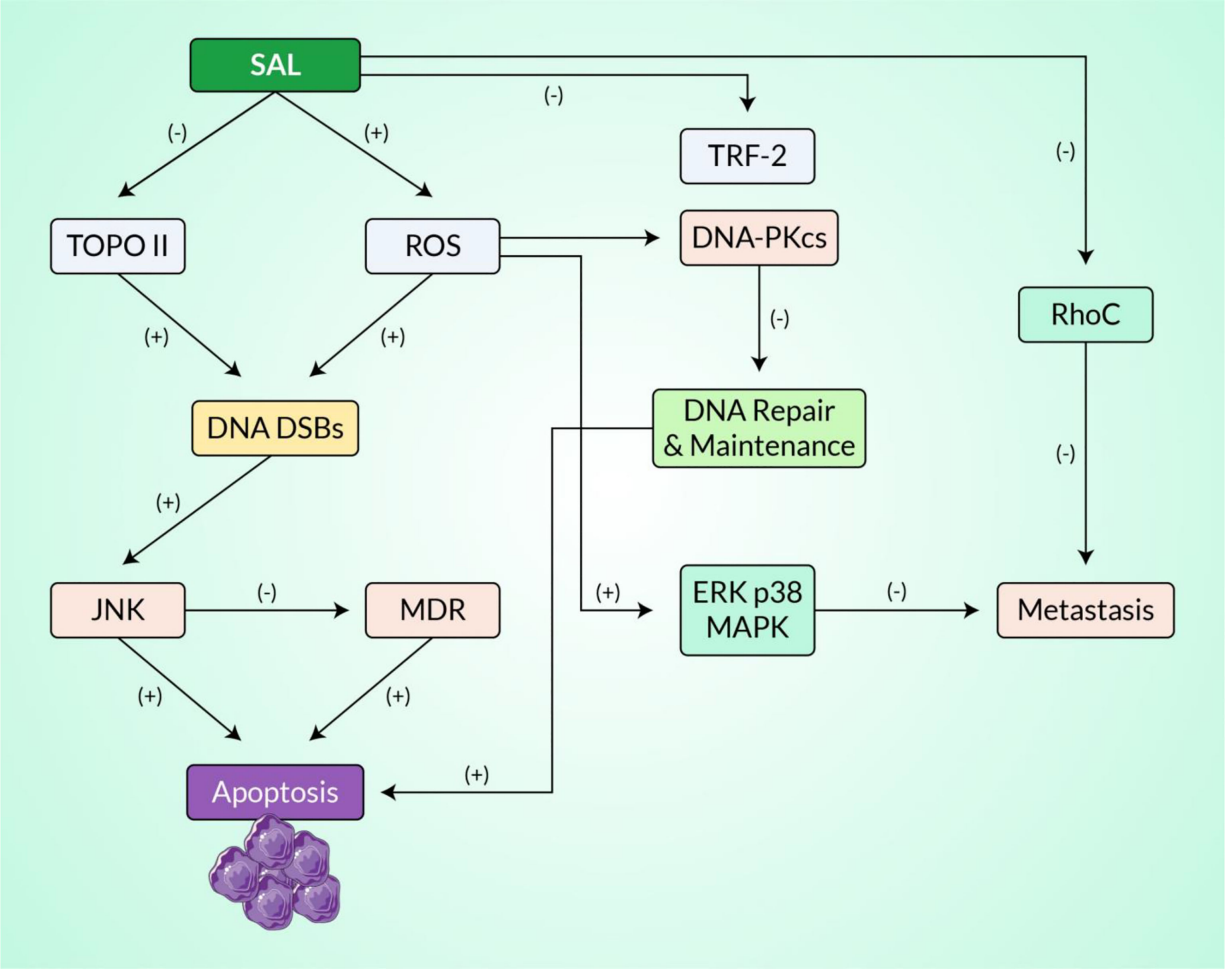
An estimated signaling pathway exhibiting the mechanisms of action of salvicine in inhibiting topoisomerase II and inducing DNA damage. All of these activities are regulated by ROS generation. Here, the *positive sign* represents stimulation or enhancement and the *negative sign* represents inhibition.

Topo II is vital for the metabolism of DNA since it plays a role in the regulation of topology during the processes of DNA replication, recombination, transcription, and in cell cycle (134). This enzyme has been recommended as a clinically crucial key route for various medical treatments such as chemotherapy against tumors, and molecules that inhibit this enzyme are the main constituents of several medical regimens (135). These Topo II inhibitors can be broadly classified based on their mode of action: Topo II poisons and its catalytic inhibitors (**Figure 4**). Topo II poisons have the capacity to sustain the reversible covalent Topo II–DNA aggregates generally known as cleavage complex, while the catalytic inhibitors follow different routes in the catalytic stages without trapping the covalent aggregates. The former are more often recommended clinically for different treatments of human cancers, but they still have some drawbacks including partial toxicities and resistance to drugs, which result in failure of treatment after the initial therapeutic promise. Furthermore, drugs synthesized from various chemical moieties, which have uniform cellular targets, show various levels of antitumor efficacy. Therefore, there is still an keen need for investigations and the design of novel anticancer molecules focusing on human Topo II (136).

Salvicine is a diterpenoid quinone molecule extracted from a Chinese herbal plant that showed therapeutic potential against a broad range of human cancerous cells when tested *in vitro* and *in vivo* in mice transfected with human tumor xenografts (46, 137). This compound showed prominent cytotoxic efficacy on MDR cells. Furthermore, it drastically regulated the lung metastatic foci of the MDA-MB-435 orthotopic xenograft without observable resistance in the initial tumor cells. Drugs that mainly act on Topo II are also recommended for hematological and solid tumors (138). They catalyze enzymatic activities that disintegrate and re-ligate DNA DSBs in order to reduce the superhelical phase of genomic DNA or unscramble linked chromosomes (139). Topo IIα and Topo IIβ genes are expressed in human cells, with Topo IIα being essential in the cancer cell cycle because it is expressed during the S and M phases of cell division and is required for DNA multiplication and chromosome separation during mitosis (99, 140). On the other hand, Topo IIβ is superfluous for cell sustainability, and its expression is not related to cell division (141). Furthermore, it is frequently entrapped by malignant transcriptional factors that provide a proliferative transcriptome to cancer cells (142). Topo II poisons are responsible for the DNA disruption to kill the growth of cancerous cells. The limitation of this mechanism of action (MOA) is that Topo II-induced DNA disruption is found in non-cancerous or normal cells. Although the NHEJ pathway can overcome DNA breakage and rescue cells, it also allows for gene deletions and recombination, both of which are malignant. Molecules such as etoposide and teniposide, which are used in therapy for secondary leukemias, have been associated with alterations in the MLL gene on chromosome 11q23 (143). In addition to this, if these cancerous cells overcome the Topo II poison dose, their treatment becomes quite tedious as they possess heterogeneous mutations. T60, on the other hand, uses a different MOA to block Topo II activity and cell growth, resulting in fewer DNA breaks and less cytotoxicity. It may also reduce the ability of other anticancer medications to maintain maximum cell growth inhibition. As a result, T60 and its analogs may have clinical benefit when used separately or in combination with other anticancer agents (144). Overall, salvicine was discovered to be a unique Topo II inhibitor with a specific spectrum of activity involving the production of ROS, increase of Topo II–DNA interaction, and blockage of Topo II-mediated DNA disruption. Therefore, elucidating the pharmacokinetic and pharmacodynamic behavior of salvicine demands more research investigations in both preclinical and clinical settings.

Furthermore, poison inhibitors block the catalytic phases of Topo II post-DNA disruption, thus enhancing the limitations of Topo II–DNA cleavage complexes, which play vital functions as genotoxic entities among cancerous cells (145). Moreover, according to the Drug Bank database, there are approx. 19 clinically accepted molecules that target Topo II, and 15 drugs are still under investigation. However, the toxicity of these drugs is widely known, with symptoms including myelosuppression and initiation of secondary cancers (146). A unique morphological feature of these drugs is the occurrence of a planar moiety that intercalates DNA. The connecting pathways of such ligands show unique bipartite association when the planar section interacts with the DNA, while the other actively functional moieties of these drugs aggregate with proteins (147). The first inhibitor co-crystallized with the Topo II protein was etoposide (PDB ID: 3QX3); therefore, most molecular docking studies were based on this structure to emphasize on model protein−ligand interactions.

## ROS-Induced and Topo II-Dependent DNA Damage Response and Apoptosis Mediated by Salvicine

Topo II poisons are well known to possess anti-proliferative activity by inducing DNA DSBs, which can trigger DNA reactivity and ultimately apoptosis. It is noteworthy that salvicine effectively increases the intracellular ROS production and thereby induces DSBs through NAC (33). NAC also prevents the generation of salvicine-induced Topo II–DNA cleavable complexes and the growth suppression of salvicine-treated JN394top2-4 yeast cells, suggesting that Topo II is a target of salvicine-induced ROS (54). Heat treatment that reversed the salvicine-trapped DNA–Topo II cleavage complex also reversed the accumulation of DNA DSB, revealing that salvicine-induced DNA damage was caused by ROS and was mediated by Topo II. DNA intercalators (e.g., doxorubicin), enzyme binders (e.g., VP16), DNA lesions (e.g., abasic sites), or oxidative stress can all disrupt the DNA–Topo II breakage and/or reunion reaction, resulting in Topo II-mediated DNA damage (35, 148). Salvicine contains a chemically active quinone moiety, and the majority of anticancer drugs containing quinones are believed to stimulate ROS as part of their anticancer potential. Salvicine contributes to the entire biological implications of salvicine therapy, such as DNA DSBs, apoptosis, and tumor cell cytotoxicity, by generating ROS to control Topo II-mediated DNA damage (35, 36).

Salvicine-induced DNA DSBs activate ATM and ATR kinases, as well as histone H2AX phosphorylation, in A549 lung cancer cells, which has been widely described in DSB-induced cellular responses (149, 150). Salvicine inhibits the catalytic subunit of DNA-dependent protein kinase (DNA-PK), but not the Ku70 or Ku86 subunits, which is surprising. In MCF-7 cells, salvicine treatment decreases DNA-PK activity, which could be due to a decrease in the (DNA-PK) protein. DNA-PK is made up of around 450 kDa DNA-PKcs and two smaller Ku subunits (Ku70 and Ku86), and it is an important element of the NHEJ pathway, which is the most common mechanism of DSB repair (including Topo II-medicated mammalian DNA repair) (151, 152). Salvicine damages the DNA at the same time, disrupting the DNA repair route that could improve its therapeutic effectiveness and overcome DNA repair resistance. NAC pretreatment abrogates the effects of salvicine on the protein level and the DNA-PK activity, which suggests that salvicine-induced DNA damage and repair involves ROS generation. The mechanism of salvicine-induced ROS on Topo II and DNA-PK provides new insights into the broad range of biological functions of ROS (34).

**Figure 5:**
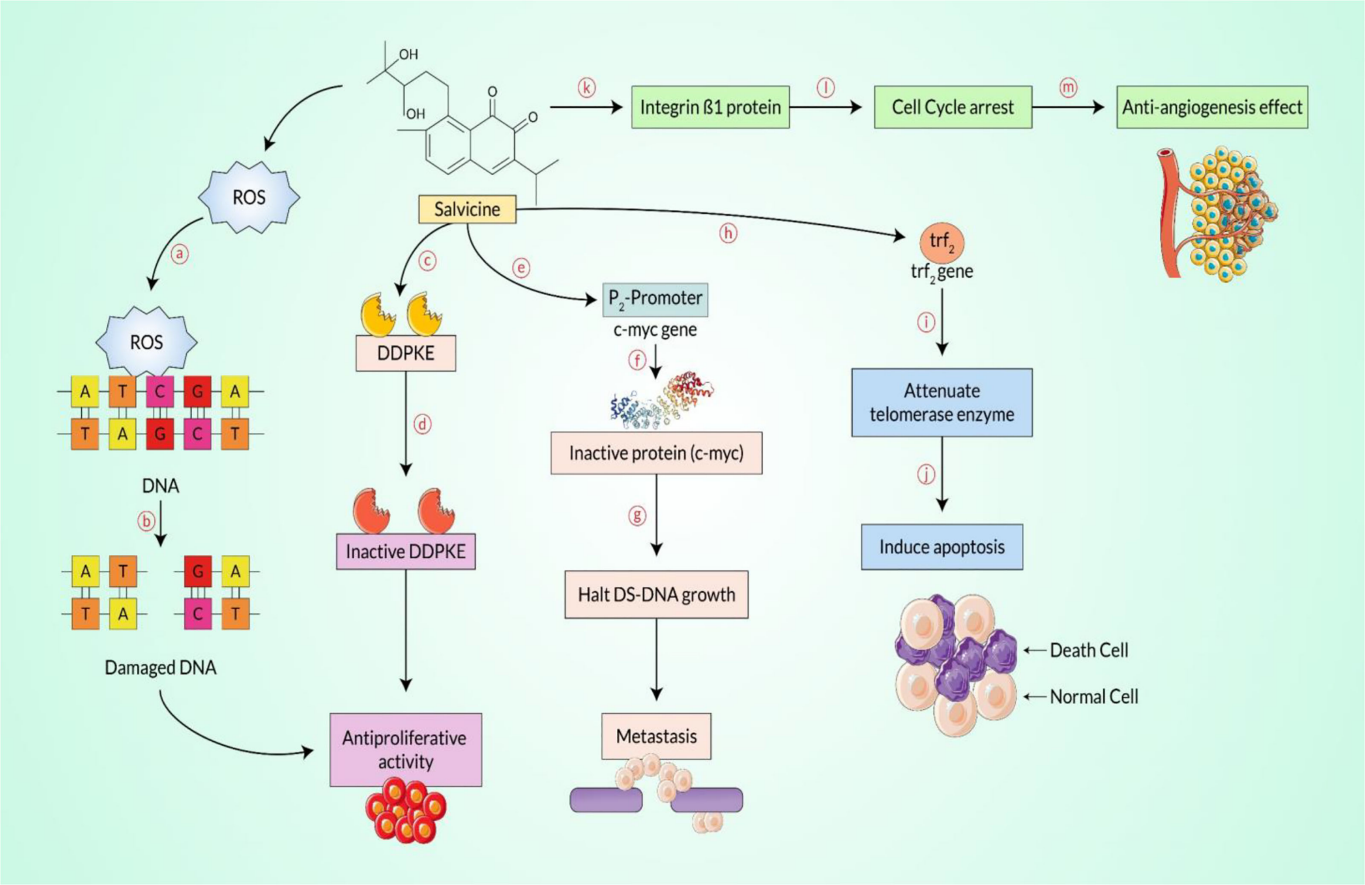
Salvicine-mediated anti-proliferative and/or anticancer effect pathway. As an initial step, salvicine produces reactive oxygen species (ROS) and binds to the double-strand (DS)-DNA moiety, the cysteine residue (*a*). At the same time, it enables disruption of the double-strand DNA (*b*), additionally inhibiting the rapidly proliferating cells, including cancerous cells. Salvicine directly interacts with the DNA-dependent protein kinase enzyme (DDPKE) (*c*), which is why the inactive enzyme functions with the inhibition of cellular growth (*d*), halting the rapidly propagating cancerous cells. On the contrary, the c-myc protein plays a key regulatory role during DS-DNA growth, but it directly binds with the promoter region of the *c-myc* gene (*e*) and interestingly yields a malfunction enzyme (*f*), which damages the DS-DNA (*g*) and subsequently downregulates metastasis. However, telomere repeat binding factor-2 is synthesized by the *trf2* gene that plays a fundamental role in normal cells, and salvicine binds to the *trf2* gene (*h*) and shuts down the expression of the telomerase protein (*i*) that induces apoptosis (*j*), which mediates the anticancer effect. Finally, salvicine disrupts the cell–extracellular matrix adhesion protein, mainly integrin β1 (*k*), which is involved in cell cycle arrest (*l*) and hinders neoangiogenesis (*m*) and even cancer metastasis.

Salvicine causes DNA DSBs in human promyelocytic leukemia HL-60 cells and MCF-7 breast cancer cells (39). The findings of a study using a genetic yeast system demonstrated that DNA damage is strongly associated with cell growth suppression, indicating that Topo II is the principal cellular target of salvicine (33). Short-term salvicine treatment generated DNA damage that could be partially repaired, although early DNA breaks resulted in the induction of apoptosis in the cells. The *c-myc* oncogene region of the P2 promoter has detected preference damages; in both HL-60 and MCF-7 cells, no clear damage to DNA was detected in the 3′ region of the same gene. Salvicine reduces *c-myc* gene transcription in a dose-dependent manner while simultaneously increasing c-Jun expression (39, 53). It is possible that DNA damage can develop early in these areas, leading to growth inhibition through changes in the expression of specific genes governing gene proliferation, including *c-myc* and *c-Jun*, and eventually cell death. Notably, salvicine shut down the oncogene expression along with the induced DSB pathway. It is also worth noting that blocking of the *c-myc* gene by conducting salvicine at the MCF-7 breast cancer cell line has been clinically proven. Salvicine, in addition to inducing genomic DNA damage, has been demonstrated to cause telomere erosion and to decrease the functioning of the telomerase enzyme (39, 49). The *trf2* gene encodes the TRF2 (telomere repeat binding factor 2) protein, which is a major telomerase and plays a critical role in telomere maintenance with DDR (51, 153). Such noble compound is directly involved in blocking the overexpressed TRF2 protein *via* transcription and proteasome degradation, eventually downregulating cellular proliferation and halting cancer progression. Recent reports have proven that salvicine directly deters the function of the telomerase enzyme, at the same time inhibiting lung carcinoma on the A549 cell line. However, it also followed the disruption of the telomerase enzyme along with the initiation of apoptosis in the HL-60 cell line (human leukemia) (33, 38). The work of Zhang et al. (52) demonstrated that salvicine blocked the function of integrin β1, which plays a crucial role in cell–ECM adhesion and maintains the signaling cascade reaction, along with regulation of cell proliferation, cellular activation, and homeostasis. Blocking the integrin protein directly halts neoangiogenesis and protects the human body from a metastatic cancer state, with both *in vivo* and *in vitro* reports also showing proof of the significant anti-angiogenesis effect of salvicine on A549 cells (represented in **Figure 5**). Overall, salvicine produces ROS, which alters DNA damage (Topo II-mediated), leading to its broad physiological effects such as cytotoxicity, DSBs, and apoptosis in cancer cells. More clinical studies should be conducted to validate the mechanisms of ROS-induced and Topo II-dependent DDR and apoptosis of tumor cells mediated by salvicine.

## Anticancer Perspective

Salvicine inhibited the growth of a variety of human tumor cells *in vitro* and in mice with human tumor xenografts (154). In particular, it exhibited superior anti-carcinogenic activity against stomach and lung cancer cells. The potential of salvicine to cause apoptosis in K-562 and SGC-7901 cells was also discovered to be connected with its anticancer therapeutic activity (64). Salvicine exhibited substantial anticancer effects against S-180 sarcoma and Lewis lung cancer in mice, as well as in human lung cancer xenografts A-549 and LAX-83 (65). In this section, we exhibit both the clinical and experimental outcomes of salvicine as a potential anticancer agent in several types of cancer cell lines and in *in vivo* research models.

### Leukemia

Qing et al. investigated *in vitro* the cytotoxic effects of salvicine in various cell lines isolated from hematopoietic and solid malignancies. Apart from salvicine in K562 cells, the HL-60 and U-937 cell lines were grown in the presence of etoposide (VP16) and vincristine (VCR). Salvicine and VP16 showed comparable efficacy (IC_50_), with VCR being the most cytotoxic compound. K562 cells were grown in higher salvicine concentrations and in a steady drug concentration at increasing time intervals, establishing the drug concentration and time dependence of cell inhibition. After prolonged exposure (more than 24 h) to salvicine, cells tend to arrest in the G1 phase, expressing an inability to duplicate their genetic material (46). Qing et al. reconfirmed these effects of salvicine on K562 cells and further documented the induction of apoptosis. Specifically, they highlighted the structural characteristics of apoptosis such as DNA fragmentation in multiples of 180–185 bases and condensed nuclei in a concentration- and time-dependent manner (155). Unpublished data also revealed that DNA–Topo II was inhibited by salvicine. Meng et al. took a step further in order to shed light on the molecular events regarding salvicine-mediated cytotoxicity and apoptosis induction (74). HL-60 is a cell line in which amplified c-myc drives carcinogenesis, implying that chromatin in the specific loci is relaxed. This renders the *c-myc* gene more accessible to topoisomerase II and prone to salvicine-mediated DSBs. At least the P2 promoter is predisposed to salvicine-induced DSB triggering apoptosis (39). The lack of correlation between growth retardation and the damage on the P2 promoter suggests that there are other crucial loci that need to be investigated. Contrary to *c-myc*, the transcription factors c-Fos and c-Jun were upregulated. c-Fos and c-Jun are implicated in signaling pathways regulating cellular proliferation, differentiation, and apoptosis (118, 156). Salvicine-induced apoptosis in HL-60 cells was studied by Liu et al., who found that telomerase played a role. They documented an inhibition of telomerase activity within the first 2 h, while the expression of hTR (hemolytic transfusion reaction) remained stable and those of TP1 (transition protein 1) and hTERT (telomerase reverse transcriptase) decreased at 6 h, assuming that a posttranslational mechanism is responsible. Protein phosphatase 2A (PP2A) was found to be upregulated, while okadaic acid, a protein phosphatase inhibitor, in co-culture with salvicine profoundly inhibited apoptosis. Conclusively, these data suggest that the stimulation of PP2A has negative effects on the activity of telomerase. There are no convincing data to implicate any caspase in the activation of PP2A despite the fact that caspase-3, in contrast with caspase-1, which remained stable, was found to be time- and dose-dependently upregulated (38). Qing et al. reported on the effects of actinomycin D (AcD) as an add-on to salvicine in K562 cells. The rationale was the documented cytotoxic and apoptotic effects of AcD in leukemic cell lines (157). Interestingly, despite the synergistic inhibition of cell growth, AcD totally suppressed the salvicine-induced apoptosis (43). By definition, the chemotherapeutic regimen should induce apoptosis since the other mechanisms of cell death elicited unwanted immune responses. Miao et al. investigated the effect of salvicine on MDR cell lines. It appears that, in the K562/A02 cell line, salvicine outweighed the effects of VCR, doxorubicin, and VP16. It also potently induced apoptosis, increasing caspase-1 and caspase-3 and decreasing bcl-2. Additionally, the encoding gene of P-gp was downregulated, in contrast to the MRP (multiple resistance proteins) and LRP (lung resistance proteins) genes (44). With regard to the downregulation of the *mdr-1* gene, Miao et al. documented a pathogenic signaling pathway. Specifically, they noticed that, at the gene and protein levels, the expression of c-Jun precedes that of P-gp. Antisense oligodeoxynucleotides interfere with the translation of hybridizing messenger RNA (mRNA) in order to prevent ribosome binding. When c-Jun AcD applied, the expression levels of c-Jun protein, mdr-1 mRNA, and P-gp were found to be decreased. In addition, c-Jun AcD diminished apoptosis and cellular growth. Collectively, Miao et al. provided sufficient evidence that the transcription factor c-Jun regulates the expression of the *mdr-1* gene in salvicine-treated K562/A02 cells (158). In conclusion, vitamin C might add a synergistic effect to the effects of salvicine. Cai et al. demonstrated a shift toward the production of ROS in the K562 and MDR cell lines, in parallel with the exhaustion of glutathione (GSH). Growth inhibition was responsive to the administration of antioxidants, e.g., NAC, GSH, and catalase, and the H_2_O_2_ scavenger. Furthermore, NAC inhibited the salvicine-mediated DNA damage, the subsequent trigger of apoptosis, and the activation of c-Jun and its aforementioned downstream effects. Conversely, H_2_O_2_ and vitamin C enhanced the effects of salvicine regarding cell growth inhibition and apoptosis induction in both K562/A02 and the parental cell lines (45).

### Gastric Carcinoma

Zhang et al. highlighted the signs of growth inhibition in SGC-7901, a gastric carcinoma cell line extracted from the pathologic lymph node of a patient with stage 4 metastatic disease (48, 159). Qing et al. exposed the SGC-7901 and MKN-28 cell lines to salvicine, VCR, and VP16. The IC_50_ values for VCR and VP16 in SGC-7901 cells exceeded 100 μmol/L, while that of salvicine was near to 7.8 μmol/L. The results of the MKN-28 cell line showed the same trend, signifying a nearly 10-fold greater potency over VCR and VP16 (46). SGC-7901 cells were exposed to increasing doses of salvicine for 24 h, establishing a dose-dependent inhibition of cellular growth, as revealed by Qing et al. Morphological signs of programmed cell death were evident and became more apparent when cells were exposed to higher drug concentrations (47).

### Lung Adenocarcinoma

Indications of cytotoxicity against lung cancer cell lines were documented by Qing et al., specifically treatment of the A549, SPCA-4, NCI-H23, and NCI-H522 cell lines with salvicine, VCR, and VP16. It was demonstrated that salvicine caused more damage in cancerous cell lines than did the classical chemotherapeutic agents (46). Miao et al. delineated the characteristics of the A549 cell line. This cell line exhibited significant resistance to salvicine and antimetabolites, while alkylating and platinum agents showed nearly the same sensitivity as that in the parental cell line. The authors reported a dose-dependent induction of apoptosis in the higher dose spectrum than in the parental A549 cells, which is another indication of resistance to salvicine. In cultures, A549/salvicine cells showed a more diffuse growth pattern, with longer doubling time than that in the parental cells. At the molecular level, p53 and Bax were downregulated in A549 cells, without enhancement of the genes regulating drug resistance (50). Liu et al., after elucidating the implication of telomerase in HL-60 leukemic cells, investigated the role of telomerase in A549 lung adenocarcinoma cells. At a low dose for longer time intervals, salvicine was the most potent telomerase inhibitor among those most commonly used and showed the unique characteristic of inhibiting telomerase when administered at a high dose for a short duration. In the range from 25 to 50 μM, salvicine dose-dependently inhibited telomerase, and increasing the treatment duration with a salvicine dose of 50 μM further reduced the enzyme’s activity. Additionally, salvicine did not appear either to interfere with telomerase directly or with the transcriptional expressions of hTERT, hTP1, and hTR, the subunits of telomerase. For example, in the HL-60 cell line, okadaic acid reversed the salvicine-induced telomerase downregulation, implying the occurrence of posttranslational telomerase modifications (49). Another research group investigated the effects of TRF2 on DNA damage repair. In addition to the salvicine-induced DSBs and telomere damage, the authors documented alterations in TRF2 function. Experimenting with *trf2* gene enhancement and transcriptional silencing, they also recorded the fundamental effects of TRF2 on the protection of genomic material from salvicine damage. They also reported that these effects were mediated by ATR, even though both ATM and ATR were overexpressed in salvicine-mediated damage. Finally, Zhang and his research collaborators, taking into consideration the anti-integrin effects of salvicine that emerged in the literature, explored the potential anti-metastatic activity of salvicine. They documented a dose-dependent inhibition of both A549 cells and human microvascular endothelial cells (HMECs). In HMECs, salvicine altered the mobility of the cells and prevented vessel tube formation. Furthermore, salvicine downregulated the expression of basic fibroblast growth factor, while the expression of vascular endothelial growth factor remained unaffected (52).

### Breast Cancer

The potency of salvicine in the MDR cell line MCF-7/ADR was discovered by Miao et al. Interestingly, they documented salvicine as being more potent than doxorubicin, VCR, and VP16. The IC_50_ values were 1.4, 4.48, 13.85, and 58.05, respectively, and the resistance factors, the IC_50_ ratios between the parental and the MDR cell lines, were 1.42, 233.19, 344.35, and 71.22, respectively (44). Lu et al. investigated the interplay between DNA damage and cellular development. They documented that 2.5 μM of salvicine was sufficient to cause DNA damage and 7.69% of growth inhibition. Higher doses of the drug were responsible for greater growth inhibition. The main type of damage was DSB, and similarly to what has been reported by Meng et al., for HL-60 cells, salvicine caused preferential damage to the P2 promoter of the *c-myc* gene, a critical transcription factor for the development of the disease (39). The *c-myc* gene was not the only gene affected. The expression of c-Jun was downregulated, and it is noteworthy that its downregulation preceded that of *c-myc*. It is not clear whether there is a causative relationship between the two events. Researchers have also documented that p53 and its downstream molecules were not implicated in the repair of salvicine-mediated DNA injury, at least in the dosage spectrum tested. As observed in other cell lines, salvicine caused a concentration-dependent induction of apoptosis. Lu et al., in order to elucidate the antineoplastic effects of salvicine, investigated whether salvicine induces the generation of ROS, with notable results. Additionally, they reported that salvicine contributed to the production of ROS. The NAC-mediated reversal of oxidative stress decreased the burden of DSBs, implying an underlying causative relationship. Salvicine-induced ROS generation affected the DNA repair pathways, interacting with DNA-PK kinase. Remarkably, pretreatment with NAC in the MCF-7 cell line reduced programmed cell death and growth inhibition, which strongly suggests that salvicine-induced ROS mediated a significant part of the effect of salvicine (54). Zhou et al., furthermore, documented the implications of ROS generation on the metastatic potential of the MCF-7 cell line. Utilizing NAC and U0126 and SB203580, which respectively inhibit ERK and p38 MAPK, the authors reported that salvicine generating ROS modified the activity of integrin β1, decreasing its migration and metastatic cell profile (55).

### Sarcoma

Salvicine has shown significant antineoplastic activity against murine S-180 sarcoma (33). *In vivo* antitumor activity studies were conducted against murine S-180 sarcoma animal models, where the results revealed remarkable activity against the models, indicating salvicine as a promising drug against sarcoma. The results are displayed in **Table 1** (48). A research study using the sarcoma S-180 murine model has demonstrated that salvicine accomplished tumor killing effects comparable to those of VP16 (48).

### Pancreatic Cancer

An experiment conducted by Yao et al. on gemcitabine-resistant SW1990 cells documented the downregulation of the deoxycytidine kinase (dCK) gene and the upregulation of ribonucleotide reductase (RR) and *mdr-1* genes. Treatment with a therapeutic dose of 4 nmol/L of salvicine did not alter the expression of *mdr-1*. At toxic concentrations, salvicine displayed a concentration-dependent decrease of *mdr-1* expression. Therefore, the research group concluded that salvicine is only able to reverse the drug resistance in pancreatic cancer cell lines when it is used at toxic concentrations (56). According to Boreddy and Srivastava, despite advancements in traditional cancer therapies such as chemotherapy, surgery, and radiation, pancreatic cancer is still the fourth deadliest cancer in the USA. As a result, phytochemicals from natural sources such as salvicine have received attention for the treatment of pancreatic cancer (160). Miao et al. found that salvicine initiates caspase-1- and caspase-3-dependent apoptosis. Although it has been assumed that caspase-1 does not cause apoptosis, recent reports have suggested that, in human pancreatic cancer cells, caspase-1 plays an important role in cell death by inducing interferon gamma (44).

### Cervical Cancer

Salvicine has anticancer properties that have been shown to positively contribute toward its efficacy to inhibit the multiplication and growth of solid tumors, such as in lung, ovary, colon, and cervical cancers (158). It has been proven to be 4.2 and 5.4 times more potent than the positive controls used, VCR and VP16, respectively. With its novel chemical properties and mode of action, salvicine, which is under phase II clinical trials, is a promising anticancer candidate that also has the benefit of low toxic side effects when used for the treatment of various cancers (161).

Cai et al. explored the role of GSH as an oxidative stress inducer in HeLa, a cervical cancer cell line. It was discovered that salvicine caused the exhaustion of intracellular GSH, modifying it directly. This led to the excessive production of H_2_O_2_, causing oxidative stress. In contrast to superoxide dismutase (SOD), NADPH oxidase inhibitors, and Trolox, NAC and GSH reversed the cell toxicity caused by salvicine. Furthermore, GSH resisted the DNA injuries and the Topo IIA inhibition induced by salvicine (57).

### Oral Carcinoma

Various chemotherapeutic drugs including cisplatin (*cis*-[PtCl_2_(NH_3_)_2_]) (CDDP), 5-fluorouracil (5-FU), and teniposide (Vm-26) have been used for the treatment of head and neck cancers, which function by inducing specific apoptotic pathways (162). The studies of Tong et al. and others have found that the apoptosis induced by 5-FU in oral squamous cell carcinoma (OSCC) is caspase-dependent, similar to that of salvicine (163, 164). Due to their effectiveness in targeting topoisomerases and causing DNA damage or tumor cell apoptosis, plant-derived compounds such as teniposide and camptothecin (CPT) have been successfully used as anticancer drugs (165). Salvicine is also a Topo II inhibitor with greater potential for use as an anticancer drug as it possesses caspase-3-dependent apoptosis-inducing properties. A study by Miao et al. (44) on the effects of salvicine on three different caspases showed that it activated caspase-1 and caspase-3 proportionally to the dose administered and the duration of treatment. Miao et al. (44) also exposed KB/VCR, an MDR oral carcinoma cell line, to salvicine, doxorubicin, VP16, and VCR. The IC_50_ values in the parental cell line were 2.26, 0.08, 1.88, 0.004, respectively, and the resistant factors were 1.93, 106.2, 28.9, and 926.86, respectively. These results indicate that salvicine is a potent inhibitor for the KB/VCR cell line (44).

### Bladder Cancer

Heyder et al. found that the invasion ability of the T24 human bladder carcinoma cell line can be reduced by inhibiting the integrin β_1_ subunit, which results in the reduced adhesion and mobility of T24 cells (166). Zhou et al. demonstrated that salvicine is able to reduce the function of integrin β_1_ by inhibiting the binding of the cell to the ECM. As a result, salvicine showed antimetastatic efficacy that can be useful for inhibiting the integrin β_1_ of bladder cancer and decreasing the adhesion and mobility of T24 cells (55).

### Prostate Cancer

Miao and Ding found that salvicine was able to increase the expression of c-Jun and decrease the expression of P-gp by inhibiting the expression of the *mdr-1* gene. It was also clear from the study that an increase in the c-Jun level is required for the downregulation of P-gp by salvicine (158). In another study, Wartenberg et al. stated that ROS were able to downregulate the expression of P-gp and activate c-Jun N-terminal kinase (JNK) in prostate tumors (167). From these two studies, it can be said that, as salvicine can also decrease P-gp expression *via* the increased c-Jun level, it might be effective against prostate cancer as well (158, 167). Another study demonstrated that extracts from several *Salvia* species were able to show growth inhibitory activity against the prostate cancer cell line MDA Pca2b. Although the authors did not use *S. prionitis* Hance, the ability of the other species certainly showed the potential of salvicine to work against prostate cancer cells (168). Zhou et al. revealed that salvicine was able to activate ERK1/2 by triggering ROS generation (55). Chen et al. found that shikonin can reduce the mobility of prostate cancer cells by using the ROS–ERK1/2 pathway, similarly to salvicine. Therefore, salvicine might have the potential to work similarly against prostate cancer (169).

### Anti-Proliferation Activity *via* the ROS-Dependent p38 MAPK Pathway

Salvicine-induced ROS are involved in several cellular functions including DNA damage, mitigating multidrug resistance, Topo II inhibition, cell adhesion inhibition, and anti-metastatic activity (75, 77). Without affecting the growth of the primary tumor, salvicine effectively suppressed the lung metastatic foci of MDA-MB-435 orthotopic xenograft (75). It is widely recognized that cell–cell interactions between cancer cells and the endothelium of distant tissues aid in the progression of cancer metastasis (170). Cell adhesion molecules such as selectins and integrins are the key players in the cell–cell interaction of tumor cells that results in tumor progression and metastasis (171, 172). Transmembrane proteins known as integrins bind to the ECM, assemble at the binding site, and initiate focal adhesion by attracting cytoplasmic proteins such as FAK, c-Src, and paxillin to the binding site (173, 174). Since cell–cell interaction is a fundamental step in the metastatic cascade, disruption of this interaction by the downregulation of the cell adhesion molecules could be a potential therapeutic approach to halting metastasis. Salvicine was found to significantly influence the cell adhesion genes involved in tumor metastasis. Human breast cancer cells treated with salvicine showed lower levels of genes for integrin a3, integrin a6, integration E, integrin b3, integrin b5, integrin b8, paxillin, and FAK.

**Figure 6:**
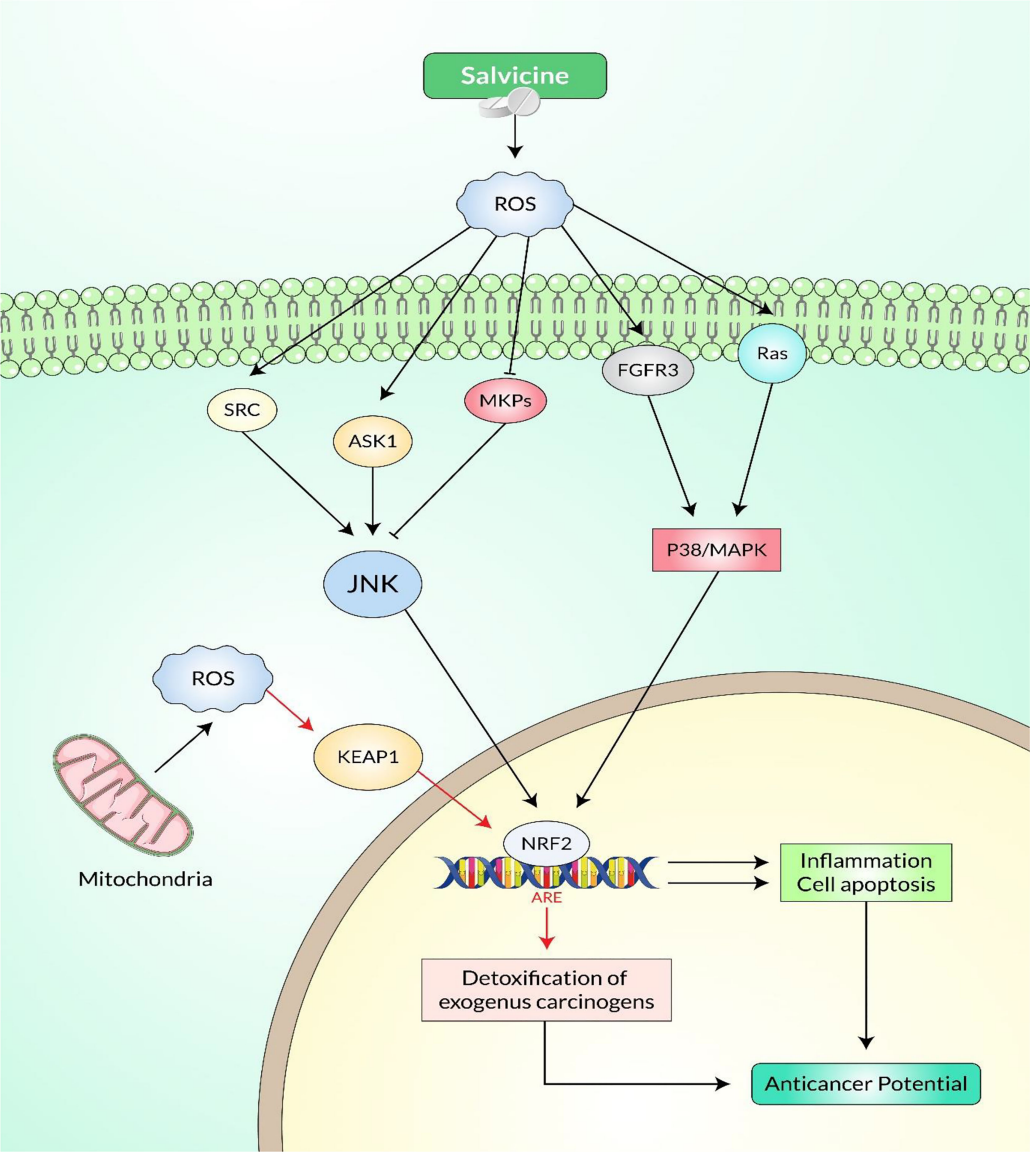
Cellular signaling pathways involved in salvicine-induced anticancer potential *via* the p38 MAPK and c-Jun N-terminal kinase (JNK) cascades.

The *in vitro* anti-metastatic efficacy of Salvicine on MDA-MB-435 orthotopic xenograft is intimately associated with Rho-dependent pathway. (75). The effect of salvicine on integrin-mediated cell adhesion has been investigated to determine its underlying anti-metastatic mechanism. Salvicine inhibited cell adherence to fibronectin and collagen in human breast cancer MDA-MB-435 cells, reduced the fibronectin-dependent establishment of focal adhesion, and disrupted the actin stress fiber networks, resulting in a rounded cell shape in the test subjects. Moreover, salvicine dephosphorylated FAK and paxillin, resulting in a downregulated integrin β1 ligand affinity, clustering, and signaling. Conversely, the anti-inflammatory drug salvicine induced an increase in the activity of ERK and p38 MAPK. Notably, U0126 and SB203580, which are inhibitors of MAPK–ERK1/2 and p38 MAPK, respectively, abolished the effect of salvicine on integrin-mediated cell adhesion (**Figure 6**) (55). According to evidence, the p38 MAPK and ERK pathways were activated in response to ROS, which raises the question whether salvicine induced ROS, which in turn activated the ERK and p38 MAPK pathways (175, 176). It has been demonstrated that the addition of NAC, a ROS scavenger, to MDA-MB-435 cells reversed the salvicine-induced activation of the p38 MAPK and ERK pathways, which facilitated the formation of integrin-mediated cell adhesion and the promotion of metastasis in cancer cells (55). Together, the results exhibited that the anti-metastatic action of salvicine on cancer cells was mediated by the ROS-triggered p38 MAPK and ERK pathways.

### Anti-Metastatic Activity *via* the Rho-Dependent Pathway

Metastasis is the process by which cancer spreads and eventually colonizes from the primary mass to distant organs. The key factors in metastasis include the microenvironment of the host tissue, i.e., angiogenesis, signals from the autocrine, paracrine, or the endocrine pathways, and the interconversion capability of cellular identities, i.e., epithelial-to-mesenchymal (EMT) and mesenchymal-to-epithelial transitions (MET) (177). EMT is in charge of local invasion, intravasation, and extravasation in metastasis, whereas MET is in charge of the recapitulation of the initial mass at distant locations in the disease progression process (178).

Migration, invasion, and adhesion are the primary stages of cancer metastatic progression. It is essential for cancer metastasis because it stimulates cell proliferation and integrin interactions, both of which are necessary for motile function and the survival of cancer cells. At the cytosolic and extracellular sites, cell detachment depends on both mechanical forces and protease-mediated cleavage. Migration occurs *via* two different mechanisms: a protease-dependent mesenchymal cell migration and a protease-independent amoeboid cell migration, which is faster than mesenchymal cell migration (179).

According to Lang et al., lung metastasis due to human breast cancer is inhibited by the anti-metastatic action of salvicine *via* the Rho-dependent pathway. Rho proteins are essential regulators of cell motility, and their overexpression is intimately correlated with metastasis. When administered in a dose- and time-dependent manner, salvicine decreased the expression of RhoC without changing the levels of RhoA, and it further decreased the level of RhoC protein in primary tumors. There are two types of G-protein-coupled receptors (G12/G13) that control how RhoA and RhoC move from the cytosol to the membrane. Lysophosphatidic acid (LPA) encourages the movement of both of these receptors. Salvicine effectively antagonizes LPA in a time- and dose-dependent manner, but independent of G12/13. Salvicine also reduces the expression of active RhoC without affecting RhoA due to its different regulatory mechanism. Additionally, salvicine significantly impairs Rho-induced stress fiber formation, impairing cell adhesion and motility and downregulating the expression of genes involved in this pathway, including integrins, fibronectin, FAK, paxillin, and RhoC (75). It was later discovered that the RhoA protein is a downstream effector of ROS-induced cytoskeleton disruption in a separate study by Zhou et al. The production of ROS caused the hydrolysis of GTP-bound RhoA, which promoted cell adhesion. Because NAC pretreatment reverses salvicine-induced damage and negatively regulates cell adhesion, salvicine drastically lowered the level of GTP-bound RhoA and destroyed the actin stress fiber networks that are critical for cell adhesion (55). Salvicine stimulated p38 MAPK and ERK by causing ROS production, which allowed inactivation of the activity of integrin β1 and retardation of cell adhesion. Overall, the anti-metastatic action of salvicine is linked to the ROS-activated p38 MAPK and Rho-dependent signaling cascade. The connection between these two signaling cascades warrants additional investigation.

### Mitigating the P-Glycoprotein-Mediated Multidrug Resistance of Chemotherapeutic Agents

Multidrug resistance is described as the ability of a tumor cell to survive and develop resistance to a wide variety of anticancer treatments. It is one of the vital reasons for the failure of cancer therapies that affect patients suffering from a variety of cancers (180, 181). When tumor cells resist anticancer drugs, the *mdr-1* gene and its product, P-gp, are overexpressed. Drugs can be expelled from cells by P-gp, an ATP-binding cassette (ABC) transporter with the potential to do so (182, 183). P-gp can attach to various anticancer drugs such as vinblastine, taxol, and doxorubicin, which causes the efflux of drug molecules into the extracellular space. Consequently, there is decreased accumulation of anticancer drugs in cells and increased resistance of cancer cells to the therapy (184, 185). The damaging role of P-gp in anticancer regimens points out a constant need for effective cytotoxic P-gp inhibitors to mitigate P-gp-associated multidrug resistance in order to improve patient outcomes.

Studies have shown that salvicine possesses cytotoxic properties against a variety of MDR tumor cells *via* the downregulation of P-gp expression. Salvicine also exhibited cytotoxicity against the MDR sub-lines K562/A02, KB/VCR, and MCF-7/ADM, with IC_50_ values (1.55, 4.50, and 1.40 µm, respectively) quite close to those (0.87, 2.26, and 2.61 µm) of the corresponding parental cell lines: K562, KB, and MCF-7, respectively. In MDR K562/A02 cells, salvicine downregulated the expressions of *mdr-1* and P-gp in a dose-dependent manner (44). It was found that salvicine suppressed the expressions of *mdr-1* and P-gp by stimulating the expression of the *c-Jun* gene in MDR K562/A02 cells. Salvicine stimulated JNK phosphorylation, which enhanced the expression of c-Jun; in turn, the activated c-Jun bound to the activator protein-1 (AP-1) target element in the *mdr-1* gene promoter region, which resulted in the decreased expressions of *mdr-1* mRNA and P-gp in MDR K562/A02 cells (158). Further study was carried out to elucidate the role of salvicine-induced ROS on P-gp expression to circumvent multidrug resistance. Salvicine produced ROS equally in both MDR K562/A02 and parental K562 cell lines (45). Pretreatment of MDR K562/A02 cells with NAC significantly reversed the salvicine-induced P-gp downregulation and JNK phosphorylation. Collectively, salvicine-induced oxidative stress increased the phosphorylation of JNK, resulting in the activation of c-Jun. The activated c-Jun enhanced the expression of *c-Jun*, suppressed the transcription of *mdr-1*, and activated pro-apoptotic pathways, leading to a reduced *mdr-1* expression and apoptosis. Overall, these results suggest the potential of salvicine to overcome multidrug resistance and promote P-gp downregulation *via* ROS generation.

## Conclusion and Future Perspectives

Specifically, salvicine is a novel Topo II toxin that binds to the ATPase domain of Topo II, increasing DNA–Topo II binding while simultaneously decreasing DNA relegation and ATP hydrolysis. Salvicine showed more potency than VCR and VP-16 at killing 12 types of solid tumor cells than they are at killing healthy cells. The potential of salvicine to cause apoptosis in K-562 and SGC-7901 cells was discovered to be connected to its anticancer activity. Additionally, salvicine induces DNA damage, bypassed by P-glycoprotein of multidrug resistance mechanism, and prevents tumor cells from sticking to each other. In MCF-7 cells, it increased intracellular ROS generation and caused significant DNA DSBs. NAC reduced the salvicine-induced ROS increase and reversed the subsequent DNA breakpoints (DSBs). It mostly inhibited pre-strand Topo II-mediated DNA re-ligation and showed little effect on the catalytic activities of the cleavage complex. Interestingly, salvicine had no effect on pBR322 relaxation mediated by Topo I, which suggests that its Topo II activity is quite selective. The Topo II catalytic cycle may be broken down into six distinct phases, each with its own mode of action. In the intended concentration range, salvicine failed to intercalate into DNA. Topo II is attacked by the antineoplastic-induced ROS, resulting in DNA DSBs. As the first experimental evidence for the role of ROS in DNA-PKcs modulation, salvicine and NAC prevented salvicine-induced cell death and cytotoxicity in MCF-7 cells. Salvicine inhibited the activity of DNA-PK, a key component of the NHEJ repair process. The downregulation of a catalytic subunit of DNA-PK might be due to one mechanism. IBC, BEL-7402, HO8910, and HCT116 nude xenografts showed no growth inhibitory effects when exposed to salvicine. All of these activities are reliant on ROS in certain ways, and the salvicine-elicited ROS appear to play a significant role in ROS generation and participation in the anticancer activity of salvicine. Salvicine can thus be employed as an anticancer medication candidate and a tool for exploring the intricate roles of ROS in the physiological functioning of tumor cells in diverse disorders, which will provide useful information for future clinical trials.

Our findings in this work suggest that salvicine-induced ROS disrupted Topo II and DNA-PK simultaneously in cultured tumor cells, affecting two components of DNA damage and repair and accounting for at least some of its anticancer effects. This is due to Topo II being able to attach to ellipticine and VP16 in the absence of DNA, according to previous research. The drug binding sites have yet to be identified. We also attempted to focus out the multidrug resistance against the cancer effects of salvicine, which are used in cancer treatments. These findings suggest that targeting certain DNA repair proteins might help enhance the existing DNA-damaging anticancer medicines and lead to the development of novel agents or treatments for cancer based on salvicine.

In this review study, we have broadly demonstrated the complicated activities of ROS in various physiological processes in tumor cells to aid in the creation of crucial DNA repair proteins or anticancer drugs by permitting the logical design of salvicine-like anti-Topo II compounds. We have exhibited that salvicine possessed potent anticancer effectivity against several cancer research models. However, further research studies are needed to evaluate its anticancer potential and its synergistic effects in combination with several conventional cancer drugs. Therefore, it can be concluded that, after a number of *in vitro* and *in vivo* clinical studies in cancer research models, salvicine can be developed as an alternative therapeutic option for cancer treatment.

## Author Contributions

DD, PB, and MMH: Research idea development and conceptualization. DD, MMH, PB, SP, RR, ST, MB, MJI, FAA, EMA, MF, SKB, and PP: Writing and main draft preparation. DD, PB, MMH: Writing, reviewing, and editing. TIR: Drawing of figures. SB, MAR, and BK: Visualization and supervision. BK: Project fund. All authors contributed to the article and approved the submitted version.

## Funding

This research was supported by the Basic Science Research Program through the National Research Foundation of Korea (NRF) funded by the Ministry of Education (NRF-2020R1I1A2066868), the National Research Foundation of Korea (NRF) grant funded by the Korea government (MSIT; no. 2020R1A5A2019413), a grant of the Korea Health Technology R&D Project through the Korea Health Industry Development Institute (KHIDI), funded by the Ministry of Health & Welfare, Republic of Korea (grant no. HF20C0116), and a grant of the Korea Health Technology R&D Project through the Korea Health Industry Development Institute (KHIDI), funded by the Ministry of Health & Welfare, Republic of Korea (grant no. HF20C0038).

## Conflict of Interest

The authors declare that the research was conducted in the absence of any commercial or financial relationships that could be construed as a potential conflict of interest.

## Publisher’s Note

All claims expressed in this article are solely those of the authors and do not necessarily represent those of their affiliated organizations, or those of the publisher, the editors and the reviewers. Any product that may be evaluated in this article, or claim that may be made by its manufacturer, is not guaranteed or endorsed by the publisher.
